# ﻿Two new species of *Perenniporia* sensu lato (Polyporales, Basidiomycota) from China and two new combinations in *Crassisporus*

**DOI:** 10.3897/mycokeys.105.121858

**Published:** 2024-04-25

**Authors:** Chao-Ge Wang, Jian Chen, Hong-Gao Liu, Yu-Cheng Dai, Yuan Yuan

**Affiliations:** 1 State Key Laboratory of Efficient Production of Forest Resources, School of Ecology and Nature Conservation, Beijing Forestry University, Beijing 100083, China; 2 Yunnan Key Laboratory of Gastrodia and Fungi Symbiotic Biology, Zhaotong University, Zhaotong 657000, China; 3 Yunnan Engineering Research Center of Green Planting and Processing of Gastrodia, Zhaotong University, Zhaotong 657000, China

**Keywords:** Phylogeny, polypore, taxonomy, wood-decaying fungi

## Abstract

Phylogenetic and morphological analyses on *Perenniporia* s.l. were carried out. Phylogenies on *Perenniporia* s.l. are reconstructed with two loci DNA sequences including the internal transcribed spacer (ITS) regions and the large subunit (nLSU). Two new species from Yunnan Province, southwest China, *Perenniporiaprunicola* and *P.rosicola* in *Perenniporia* s.l., are illustrated and described. *Perenniporiaprunicola* is characterised by the perennial and resupinate basidiomata with a clay pink pore surface when fresh, a trimitic hyphal system, the presence of clavate to fusiform hymenial cystidia, ellipsoid to broadly ellipsoid basidiospores measuring 4.8–6.2 × 3.6–4.5 µm. *Perenniporiarosicola* is characterised by annual and resupinate basidiomata with a white pore surface when fresh, a dimitic hyphal system, the presence of dendrohyphidia, broadly ellipsoid to subglobose basidiospores measuring 5–5.8 × 4–5.2 μm. In addition, *Crassisporus* is a genus in *Perenniporia* s.l., in which two new combinations *Crassisporusminutus* and *C.mollissimus* are proposed. Main morphological characteristics of species related to new taxa are also provided.

## ﻿Introduction

*Perenniporia* Murrill (Polyporales, Basidiomycetes) is typified by *P.medulla-panis* (Jacq.) Donk and it is one of the species-rich genera of Polyporales. Traditionally, it is characterised by annual to perennial, resupinate, effused-reflexed to pileate basidiomata with a varied coloured pore surface when fresh, a dimitic to trimitic hyphal system with generative hyphae bearing clamp connections, variably dextrinoid and cyanophilous skeletal hyphae, ellipsoid, broadly ellipsoid to subglobose, mostly thick-walled and truncate variably dextrinoid, cyanophilous basidiospores and causing a white rot in dead angiosperm and gymnosperm woods (Ryvarden and Gilbertson 1994; Decock and Ryvarden 1999; Zhao et al. 2013a; Cui et al. 2019; Ji et al. 2023).

*Perenniporia* was established by Murrill in 1942 just with two species, *P.unita* (Pers.) Murrill (Basionym: *Polyporusunitus* Pers.) and *P.nigrescens* (Bres.) Murrill (Basionym: *Porianigrescens* Bres.), none of which was regarded as the type species (Murrill 1942). Then *P.unita* was combined into different genera by other mycologists, viz. *Fibuloporiaunita* (Pers.) Bondartsev, *Fomesunitus* (Pers.) J. Lowe and *Fomitopsisunita* (Pers.) Bondartsev (Bondartsev 1953; Lowe 1955), as well as being designated the lectotype of *Perenniporia* by Cooke (1953). Decock and Stalpers (2006) re-discussed the relationship and status of *Polyporusunitus* and *Boletusmedulla-panis* Jacq., though they are synonymous and the latter has been normally regarded as the type species of *Perenniporia* in previous studies (Donk 1960; Ryvarden 1972a; Gilbertson and Ryvarden 1987; Ryvarden and Gilbertson 1994). In addition, they demonstrated *Pol.unitus* is not a synonym of *B.medulla-panis*, the latter of which was selected as the type of *Perenniporia* (Decock and Stalpers 2006). For now, *Porianigrescens* as a synonym of *Physisporinuscrocatus* (Pat.) F. Wu, Jia J. Chen & Y.C. Dai was described from Hungary and it has a perennial basidiomata, erubescent pores (white when fresh, then “carneo-violaceis”, finally black), but no basidiospores data (Bresadola 1897).

Previous studies have shown that *Perenniporia* is a polyphyletic genus (Zhao et al. 2013a; Cui et al. 2019; Ji et al. 2023). Species in *Perenniporia* s.l. form seven independent clades, based on phylogenetic analysis with typical characteristics (Zhao et al. 2013a). *Hornodermoporus*, *Perenniporiella*, *Truncospora*, *Vanderbylia* etc. were derived from *Perenniporia* s.l. Especially, Ji et al. (2023) proposed 15 new genera previously addressed in *Perenniporia* s.l., based on phylogenetic and morphological analyses. *Perenniporia* s.s. contains three species, viz. *P.hainaniana* B.K. Cui & C.L. Zhao, *P.medulla-panis* and *P.substraminea* B.K. Cui & C.L. Zhao (Ji et al. 2023). Up to now, more than 120 taxa were found in *Perenniporia* s. l. (Ji et al. 2017; Liu et al. 2017; Shen et al. 2018; Cui et al. 2019; Zhao and Ma 2019; Ji et al. 2023). In addition, some species in *Perenniporia* s.l. could produce laccase (such as *P.tephropora* (Mont.) Ryvarden and *Poriellasubacida* (Peck) C.L. Zhao) and carotenoid (such as *Vanderbyliafraxinea* (Bull.) D.A. Reid) etc. applied in both biomedical engineering and biodegradation (Si et al. 2011; Churapa and Lerluck 2016; Kim and Lee 2020).

*Crassisporus* B.K. Cui & Xing Ji was proposed as a new genus (Ji et al. 2019) and it has effused-reflexed to pileate basidiomata with a mostly concentrically zonate pileal surface, a trimitic hyphal system with inamyloid or non-dextrinoid skeletal hyphae, oblong to broadly ellipsoid, slightly thick-walled basidiospores (Ji et al. 2019). Four species are included in this genus currently.

During the fungal research work on polypores, the phylogeny, based on a two loci dataset (ITS+nLSU), was carried out and two unknown species of *Perenniporia* s.l. are found from southwest China and they are illustrated and described in the present paper. In addition, two new combinations in *Crassisporus* are proposed, based on phylogenetic and morphological analyses.

## ﻿Materials and methods

### ﻿Morphological studies

The studied specimens are deposited in the Fungarium of the Institute of Microbiology, Beijing Forestry University (BJFC) and the Institute of Applied Ecology, Chinese Academy of Sciences (IFP). Morphological descriptions are based on field notes and voucher specimens. The microscopic analysis follows Miettinen et al. (2018) and Wu et al. (2022). Sections were studied at a magnification of up to 1000× using a Nikon Eclipse 80i microscope and phase contrast illumination. Microscopic features and measurements were made from slide preparations stained with Cotton Blue and Melzer’s reagent. Basidiospores were measured from sections cut from the tubes. To represent the variation in the size of spores, 5% of measurements were excluded from each end of the range and are given in parentheses. In the description: KOH = 5% potassium hydroxide, IKI = Melzer’s reagent, IKI+ = amyloid or dextrinoid, IKI– = neither amyloid nor dextrinoid, CB = Cotton Blue, CB+ = cyanophilous in Cotton Blue, CB– = acyanophilous in Cotton Blue, L = arithmetic average of spore length, W = arithmetic average of spore width, Q = L/W ratios and n = number of basidiospores/measured from given number of specimens. Colour terms follow Anonymous (1969) and Petersen (1996).

### ﻿DNA extraction, amplification and sequencing

A CTAB rapid plant genome extraction kit-DN14 (Aidlab Biotechnologies Co., Ltd, Beijing) was used to obtain DNA from dried specimens and to perform the polymerase chain reaction (PCR) according to the manufacturer’s instructions with some modifications (Shen et al. 2019; Sun et al. 2020). The internal transcribed spacer (ITS) and large subunit nuclear ribosomal RNA gene (nLSU) were amplified using the primer pairs ITS5/ITS4 and LR0R/LR7 (White et al. 1990; Hopple and Vilgalys 1999) (https://sites.duke.edu/vilgalyslab/rdna_primers_for_fungi/).

The PCR procedure for ITS was as follows: initial denaturation at 95 °C for 3 min, followed by 34 cycles at 94 °C for 40 s, annealing at 54 °C for 45 s and extension 72 °C for 1 min and a final extension of 72 °C for 10 min. The PCR procedure for nLSU was as follows: initial denaturation at 94 °C for 1 min, followed by 34 cycles of denaturation at 94 °C for 30 s, annealing at 50 °C for 1 min and extension at 72 °C for 1.5 min and a final extension at 72 °C for 10 min. The PCR products were purified and sequenced at the Beijing Genomics Institute (BGI), China, with the same primers. DNA sequencing was performed at the Beijing Genomics Institute and the newly-generated sequences were deposited in GenBank. All sequences analysed in this study are listed in Table 1. Sequences generated from this study were aligned with additional sequences downloaded from GenBank using BioEdit (Hall 1999) and ClustalX (Thompson et al. 1997). The final ITS and nLSU datasets were subsequently aligned using MAFFT v.7 under the E-INS-i strategy with no cost for opening gaps and equal cost for transformations (command line: mafft –genafpair –maxiterate 1000) (Katoh and Standley 2013) and visualised in BioEdit (Hall 1999). Alignments were spliced and transformed formats in Mesquite v.3.2. (Maddison and Maddison 2017). Multiple sequence alignments were trimmed by trimAI v.1.2 using the -htmlout-gt 0.8 -st option to deal with gaps, when necessary (Capella-Gutierrez et al. 2009).

**Table 1.** Information for the sequences used in this study.

Species name	Sample no.	Location	GenBank accession No.		References
			ITS	nLSU	
*Abundisporusfuscopurpureus*	Cui 8638	China	JN048771	JN048790	Zhao et al. (2015)
*Abundisporuspubertatis*	Dai 11927	China	KC787569	KC787576	Zhao et al. (2015)
*Abundisporuspubertatis*	Dai 12140	China	JN048772	JN048791	Zhao et al. (2015)
*Abundisporussclerosetosus*	MUCL 41438	Singapore	FJ411101	FJ393868	Robledo et al. (2009)
*Abundisporusviolaceus*	MUCL 38617	Zimbabwe	FJ411100	FJ393867	Robledo et al. (2009)
*Amylosporiahattorii*	Cui 10912	China	KX900675	KX900725	Cui et al. (2019)
*Amylosporiahattorii*	Dai 10315	China	JQ861740	JQ861756	Cui et al. (2019)
*Aurantioporiaaurantiaca*	CBS 125867	French Guiana	MH863779	MH875242	Vu et al. (2019)
*Aurantioporiabambusicola*	Cui 11050	China	KX900668	KX900719	Cui et al. (2019)
*Citrinoporiacitrinoalba*	Cui 13615	China	MG847215	MG847224	Cui et al. (2019)
*Citrinoporiacitrinoalba*	Dai 13643	China	KX880622	KX880661	Cui et al. (2019)
*Citrinoporiacorticola*	Dai 18633	Malaysia	MT117217	MT117222	Wang et al. (2020)
*Citrinoporiacorticola*	Dai 18641	Malaysia	MT117218	MT117223	Wang et al. (2020)
*Citrinoporiacorticola*	Dai 17778	Singapore	MT117219	MT117224	Wang et al. (2020)
*Citrinoporiacorticola*	Dai 18526	Malaysia	MT117216	MT117221	Wang et al. (2020)
*Crassisporusimbricatus*	Dai 10788	China	KC867350	KC867425	Cui et al. (2019)
*Crassisporusleucoporus*	Cui 16801	Australia	MK116488	MK116497	Ji et al. (2019)
*Crassisporusmacroporus*	Cui 14468	China	MK116486	MK116495	Ji et al. (2019)
*Crassisporusmicrosporus*	Dai 16221	China	MK116487	MK116496	Ji et al. (2019)
***Crassisporusminutus***	Zhou 120	China	JX163055	JX163056	Unpublished
***Crassisporusminutus***	Cui 6595	China	KX081079	KX081142	Unpublished
***Crassisporusminutus***	Dai 22571	China	PP034100ª	PP034116ª	Present study
***Crassisporusmollissimus***	Cui 6257	China	JX141451	JX141461	Zhao et al. (2015)
***Crassisporusmollissimus***	Dai 10764	China	JX141452	JX141462	Zhao et al. (2015)
*Cystidioporiapiceicola*	Cui 10460	China	JQ861742	JQ861758	Zhao and Cui (2013a)
*Cystidioporiapiceicola*	Dai 4181	China	JF706328	JF706336	Cui and Zhao (2012)
*Daedaleaquercina*	Dai 12659	Finland	KP171208	KP171230	Han et al. (2015)
*Dendroporiacinereofusca*	Dai 9289	China	KF568893	KF568895	Zhao et al. (2014b)
*Dendroporiacinereofusca*	Cui 5280	China	KF568892	KF568894	Zhao et al. (2014b)
*Fomitopsispinicola*	Cui 10405	China	KC844852	KC844857	Unpublished
*Hornodermoporuslatissima*	Cui 6625	China	HQ876604	HQ876604	Zhao et al. (2014a)
*Hornodermoporuslatissimus*	Dai 12054	China	KX900639	KX900686	Cui et al. (2019)
*Hornodermoporusmartius*	MUCL 41677	Argentina	FJ411092	FJ393859	Robledo et al. (2009)
*Hornodermoporusmartius*	MUCL 41678	Argentina	FJ411093	FJ393860	Robledo et al. (2009)
*Hornodermoporusmartius*	Cui 7992	China	HQ876603	HQ654114	Zhao et al. (2014a)
*Luteoperenniporiaaustraliensis*	Cui 16742	Australia	OK642220	OK642275	Ji et al. (2023)
*Luteoperenniporiaaustraliensis*	Cui 16743	Australia	OK642221	OK642276	Ji et al. (2023)
*Luteoperenniporiabannaensis*	Cui 8560	China	JQ291727	JQ291729	Zhao and Cui (2013a)
*Luteoperenniporiabannaensis*	Cui 8562	China	JQ291728	JQ291730	Zhao and Cui (2013a)
*Luteoperenniporiamopanshanensis*	CLZhao 5145	China	MH784912	MH784916	Zhao and Ma (2019)
*Luteoperenniporiamopanshanensis*	CL Zhao 5152	China	MH784913	MH784917	Zhao and Ma (2019)
*Luteoperenniporiayinggelingensis*	Cui 13625	China	MH427960	MH427967	Cui et al. (2019)
*Luteoperenniporiayinggelingensis*	Cui 13627	China	MH427957	MH427965	Cui et al. (2019)
*Macroporialacerata*	Cui 7220	China	JX141448	JX141458	Zhao and Cui (2013a)
*Macroporialacerata*	Dai 11268	China	JX141449	JX141459	Zhao and Cui (2013a)
*Macroporiamacropora*	Zhou 280	China	JQ861748	JQ861764	Zhao and Cui (2013a)
*Macroporiasubrhizomorpha*	LWZ 20190722‐36	China	MZ578440	MZ578444	Tian et al. (2021)
*Macrosporiananlingensis*	Cui 7620	China	HQ848477	HQ848486	Zhao and Cui (2013a)
*Macrosporiananlingensis*	Cui 7541	China	HQ848479	HQ848488	Zhao and Cui (2013a)
*Microporellussubadustus*	Cui 8459	China	HQ876606	HQ654113	Ji et al. (2023)
*Microporellusviolaceo-cinerascens*	MUCL 45229	Ethiopia	FJ411106	FJ393874	Robledo et al. (2009)
*Minoporusminor*	Cui 5782	China	HQ883475	–	Zhao and Cui (2013a)
*Minoporusminor*	Dai 9198	China	KF495005	KF495016	Cui et al. (2019)
*Neoporiabostonensis*	CLZhao 2854	USA	MG491284	MG491287	Shen et al. (2018)
*Neoporiabostonensis*	CL Zhao 2855	USA	MG491285	MG491285	Shen et al. (2018)
*Neoporiakoreana*	KUC20091030-32	Korea	KJ156313	KJ156305	Jang et al. (2015)
*Neoporiakoreana*	KUC20081002J-02	Korea	KJ156310	KJ156302	Jang et al. (2015)
*Neoporiarhizomorpha*	Cui 7507	China	HQ654107	HQ654117	Zhao and Cui (2013a)
*Neoporiarhizomorpha*	Dai 7248	China	JF706330	JF706348	Zhao and Cui (2013a)
*Niveoporiadecurrata*	Dai 16637	Thailand	KY475566	OP289291	Ji et al. (2017)
*Niveoporiadecurrata*	Dai 16660	Thailand	KY475567	OP289292	Ji et al. (2017)
*Niveoporiarusseimarginata*	Yuan 1244	China	JQ861750	JQ861766	Zhao and Cui (2013a)
*Niveoporiasubrusseimarginata*	Cui 16991	China	OK642224	OK642279	Ji et al. (2023)
*Niveoporiasubrusseimarginata*	Cui 16980	China	OK642223	OK642278	Ji et al. (2023)
Perenniporiacf.dendrohyphidia	Zhou 273	China	KX900670	–	Cui et al. (2019)
*Perenniporiaeugeissonae*	Dai 18600	Malaysia	MT232518	MT232512	Wang et al. (2020)
*Perenniporiaeugeissonae*	Dai 18605	Malaysia	MT232519	MT232513	Wang et al. (2020)
*Perenniporiahainaniana*	Cui 6366	China	JQ861745	JQ861761	Zhao and Cui (2013a)
*Perenniporiahainaniana*	Cui 6365	China	JQ861744	JQ861760	Zhao and Cui (2013a)
*Perenniporialuteola*	Harkonen 1308a	China	JX141456	JX141466	Zhao and Cui (2013b)
*Perenniporialuteola*	Harkonen 1308b	China	JX141457	JX141467	Zhao and Cui (2013b)
*Perenniporiamedulla-panis*	Cui 3274	China	JN112792	JN112793	Zhao et al. (2014a)
*Perenniporiamedulla-panis*	MUCL 43250	Norway	FJ411087	FJ393875	Robledo et al. (2009)
*Perenniporianonggangensis*	GXU 2098	China	KT894732	KT894733	Huang et al. (2017)
*Perenniporianonggangensis*	Dai 17857	Singapore	MT232521	MT232515	Huang et al. (2017)
***Perenniporiaprunicola***	Dai 24280	China	PP034101ª	PP034117ª	Present study
***Perenniporiaprunicola***	Dai 24751	China	PP034102ª	PP034118ª	Present study
***Perenniporiaprunicola***	Dai 24752	China	PP034103ª	–	Present study
*Perenniporiapseudotephropora*	Dai 17383	Brazil	MT117215	MT117220	Wang et al. (2020)
***Perenniporiarosicola***	Dai 22563	China	PP034110ª	PP034123ª	Present study
*Perenniporiastraminea*	Cui 8858	China	HQ654104	JF706334	Zhao and Cui (2013a)
*Perenniporiastraminea*	Cui 8718	China	HQ876600	HQ876600	Zhao and Cui (2013a)
*Perenniporiasubstraminea*	Cui 10191	China	JQ001853	JQ001845	Zhao et al. (2014a)
*Perenniporiasubstraminea*	Cui 10177	China	JQ001852	JQ001844	Zhao et al. (2014a)
*Perenniporiasubtephropora*	Dai 10962	China	JQ861752	JQ861768	Zhao and Cui (2013a)
*Perenniporiasubtephropora*	Dai 24890	China	PP034104ª	PP034119ª	Present study
*Perenniporiasubtephropora*	Dai 25025	China	PP034105ª	PP034120ª	Present study
*Perenniporiasubtephropora*	Dai 24871	China	PP034106ª	–	Present study
*Perenniporiasubtephropora*	Dai 10964	China	JQ861753	JQ861769	Zhao and Cui (2013a)
*Perenniporiasubtephropora*	Dai 24877	China	PP034107ª	PP034121ª	Present study
*Perenniporiatephropora*	Cui 9029	China	HQ876601	JF706339	Zhao and Cui (2013a)
*Perenniporiatephropora*	Cui 6331	China	HQ848473	HQ848484	Zhao and Cui (2013a)
*Perenniporiatephropora*	Dai 25106	China	PP034108ª	–	Present study
*Perenniporiatephropora*	Dai 24849	China	PP034109ª	PP034122ª	Present study
*Perenniporiellachaquenia*	MUCL 47647	Argentina	FJ411083	FJ393855	Robledo et al. (2009)
*Perenniporiellachaquenia*	MUCL 47648	Argentina	FJ411084	FJ393856	Robledo et al. (2009)
*Perenniporiellamicropora*	MUCL 43581	Cuba	FJ411086	FJ393858	Robledo et al. (2009)
*Perenniporiopsisminutissima*	Cui 10979	China	KF495003	KF495013	Cui et al. (2019)
*Perenniporiopsisminutissima*	Dai 12457	China	KF495004	KF495014	Cui et al. (2019)
*Perenniporiopsisminutissima*	Dai 17383	Brazil	MT117215	MT117220	Wang et al. (2020)
*Perenniporiopsisminutissima*	Dai 24887	China	PP034111ª	–	Present study
*Perenniporiopsisminutissima*	Dai 24885	China	PP034112ª	–	Present study
*Perenniporiopsisminutissima*	Cui 10221	China	KX962546	KX962553	Wu et al. (2017)
*Perenniporiopsissinensis*	Dai 26477	China	PP034113ª	PP034124ª	Present study
*Perenniporiopsissinensis*	CLZhao 8278	China	OR149913	OR759768	Yang et al. (2024)
*Poriellaafricana*	Cui 8674	China	KF018119	KF018128	Zhao et al. (2015)
*Poriellaafricana*	Cui 8676	China	KF018120	KF018129	Zhao et al. (2015)
*Poriellaellipsospora*	Cui 10284	China	JQ861739	KF018133	Shen et al. (2018)
*Poriellaellipsospora*	Cui 10276	China	KF018124	KF018132	Shen et al. (2018)
*Poriellasubacida*	Dai 8224	China	HQ876605	JF713024	Zhao and Cui (2013a)
*Poriellavalliculorum*	LE 222974	Russia	KM411458	KM411474	Zmitrovich and Kovalenko (2016)
*Poriellavalliculorum*	Cui 10053	China	KF495006	KF495017	Zhao et al. (2014a)
*Rhizoperenniporiajaponica*	Cui 7047	China	KX900677	KX900727	Cui et al. (2019)
*Sparsitubusnelumbiformis*	Cui 6590	China	KX880632	KX880671	Cui et al. (2019)
*Sparsitubusnelumbiformis*	Cui 8497	China	KX880631	KX880670	Cui et al. (2019)
*Tropicoporiaaridula*	Dai 12398	China	JQ001855	JQ001847	Zhao and Cui (2013a)
*Tropicoporiaaridula*	Dai 12396	China	JQ001854	JQ001846	Zhao and Cui (2013a)
*Truncatoporiapyricola*	Cui 9149	China	JN048762	JN048782	Zhao and Cui (2013a)
*Truncatoporiapyricola*	Dai 10265	China	JN048761	JN048781	Zhao and Cui (2013a)
*Truncatoporiatruncatospora*	Cui 6987	China	JN048778	HQ654112	Zhao and Cui (2013a)
*Truncatoporiatruncatospora*	Dai 5125	China	HQ654098	HQ848481	Zhao and Cui (2013a)
*Truncosporadetrita*	MUCL 42649	French Guiana	FJ411099	FJ411099	Robledo et al. (2009)
*Truncosporamacrospora*	Cui 8106	China	JX941573	JX941596	Zhao and Cui (2013c)
*Truncosporaochroleuca*	MUCL 39726	China	FJ411098	FJ393865	Robledo et al. (2009)
*Truncosporaochroleuca*	Dai 11486	China	HQ654105	JF706349	Zhao and Cui (2012)
*Truncosporaochroleuca*	MUCL 39563	Australia	FJ411097	FJ393864	Robledo et al. (2009)
*Truncosporaohiensis*	Cui 5714	China	HQ654103	HQ654116	Cui and Zhao (2012)
*Truncosporaohiensis*	MUCL 41036	USA	FJ411096	FJ393863	Robledo et al. (2009)
*Truncosporaornata*	SP 6672	Russia	KJ410690	–	Spirin et al. (2015)
*Vanderbyliadelavayi*	Dai 6891	China	JQ861738	–	Zhao et al. (2014a)
*Vanderbyliafraxinea*	Cui 8871	China	JF706329	JF706345	Zhao et al. (2014a)
*Vanderbyliafraxinea*	Cui 8885	China	HQ876611	JF706344	Zhao et al. (2014a)
*Vanderbyliafraxinea*	DP 83	Italy	AM269789	AM269853	Guglielmo et al. (2007)
*Vanderbyliarobiniophila*	Cui 7144	China	HQ876608	JF706341	Zhao et al. (2014a)
*Vanderbyliarobiniophila*	Cui 5644	China	HQ876609	HQ876609	Zhao and Cui (2013a)
*Vanderbyliavicina*	MUCL 44779	Ethiopia	FJ411095	FJ393862	Robledo et al. (2009)
*Vanderbyliella* sp.	Knudsen 04‐111	China	JQ861737	JQ861755	Zhao and Cui (2013a)
*Vanderbyliellatianmuensis*	Cui 2715	China	JX141454	JX141464	Zhao and Cui (2013a)
*Vanderbyliellatianmuensis*	Cui 2648	China	JX141453	JX141463	Zhao and Cui (2013a)
*Xanthoperenniporiamaackiae*	Dai 8929	China	HQ654102	JF706338	Zhao and Cui (2013a)
*Xanthoperenniporiamaackiae*	Cui 5605	China	JN048760	JN048780	Zhao et al. (2013b)
*Xanthoperenniporiapunctata*	Dai 26121	China	PP034114ª	–	Present study
*Xanthoperenniporiapunctata*	Dai 26120	China	PP034115ª	–	Present study
*Xanthoperenniporiapunctata*	Dai 17916	China	MG869686	MG869688	Li et al. (2018)
*Xanthoperenniporiasubcorticola*	Dai 7330	China	HQ654094	HQ654108	Zhao and Cui (2013a)
*Xanthoperenniporiasubcorticola*	Cui 1248	China	HQ848472	HQ848482	Zhao and Cui (2013a)
*Xanthoperenniporiasubcorticola*	Cui 2655	China	HQ654093	HQ654093	Zhao and Cui (2012)
*Xanthoperenniporiatenuis*	Wei 2969	China	JQ001859	JQ001849	Zhao and Cui (2013a)
*Xanthoperenniporiatenuis*	Wei 2783	China	JQ001858	JQ001848	Zhao and Cui (2013a)
*Yuchengiakilemariensis*	LE 214743	Russia	KM411457	KM411473	Zmitrovich and Kovalenko (2016)
*Yuchengianarymica*	Dai 10510	China	HQ654101	JF706346	Zhao et al. (2013b)

ª Newly-generated sequences in this study. **Bold** = new taxa.

### ﻿Phylogenetic analyses

In this study, one combined matrix was reconstructed for phylogenetic analyses; a two loci dataset (ITS+nLSU) was used to determine the phylogenetic position of the new species. The sequence alignments and the retrieved topologies were deposited in TreeBase (http://www.treebase.org), under accession ID: 31050 (Reviewer access URL: http://purl.org/phylo/treebase/phylows/study/TB2:S31050?x-access-code=fa4d2a2edcdd53d63276b66a95c2058d&format=html). Sequences of *Fomitopsispinicola* (Sw.) P. Karst. and *Daedaleaquercina* (L.) Pers., obtained from GenBank, were used as the outgroups (Ji et al. 2023). The phylogenetic analyses followed the approach of Han et al. (2016) and Zhu et al. (2019). Maximum Likelihood (ML) and Bayesian Inference (BI) analyses were performed, based on the two datasets. The best-fit evolutionary model was selected by Hierarchical Likelihood Ratio Tests (HLRT) and Akaike Information Criterion (AIC) in MrModelTest 2.2 (Nylander 2004) after scoring 24 models of evolution in PAUP* version 4.0b10 (Swofford 2002).

Sequences were analysed using Maximum Likelihood (ML) with RAxML-HPC2 through the CIPRES Science Gateway (www.phylo.org; Miller et al. 2010). Branch support (BT) for ML analysis was determined by 1000 bootstrap replicates. Bayesian phylogenetic inference and Bayesian Posterior Probabilities (BPP) were computed with MrBayes 3.1.2 (Ronquist and Huelsenbeck 2003). Four Markov chains were run for 5 M generations (two loci dataset) until the split deviation frequency value was less than 0.01 and trees were sampled every 100 generations. The first 25% of the sampled trees were discarded as burn-in and the remaining ones were used to reconstruct a majority rule consensus and calculate Bayesian Posterior Probabilities (BPP) of the clades. All trees were viewed in FigTree v. 1.4.3 (http://tree.bio.ed.ac.uk/software/figtree/). Branches that received bootstrap support for ML (≥ 75% (ML-BS)) and BPP (≥ 0.95 BPP) were considered as significantly supported. The ML bootstrap (ML) ≥ 50% and BBP (BPP) ≥ 0.90 are presented on topologies from ML analysis, respectively.

## ﻿Results

### ﻿Molecular phylogeny

**Figure 1.**
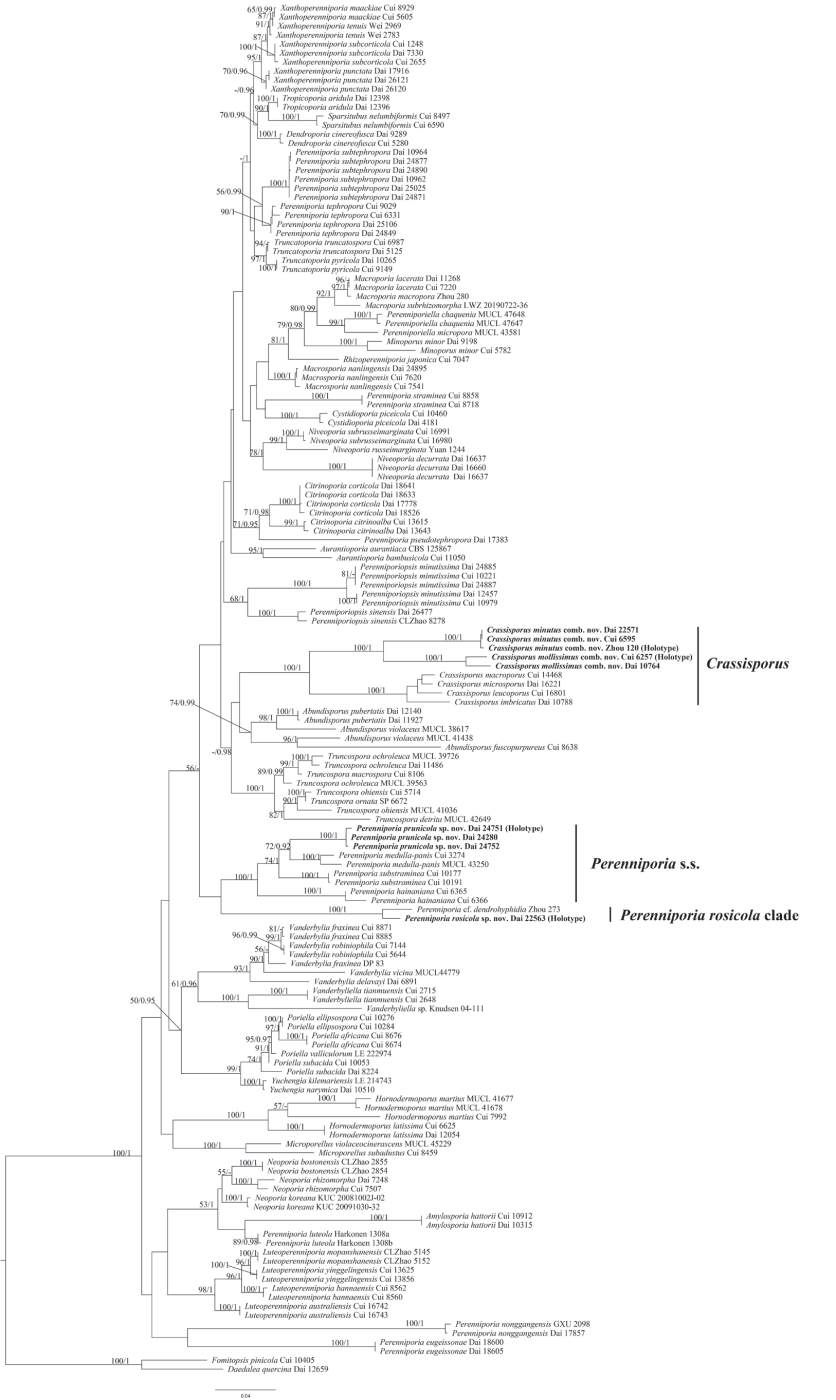
ML analysis of *Perenniporia* s.l. based on dataset of ITS+nLSU. ML bootstrap values higher than 50% and Bayesian posterior probabilities values more than 0.90 are shown. New taxa are in bold.

The combined two loci dataset (ITS+nLSU) included sequences from 152 samples representing 80 taxa. The dataset had an aligned length of 2156 characters, of which 1385 (64%) characters were constant, 147 (7%) were variable and parsimony-uninformative and 624 (29%) were parsimony informative. The phylogenetic reconstruction performed with Maximum Likelihood (ML) and Bayesian Inference (BI) analyses for one combined dataset showed similar topology and few differences in statistical support. The best model-fit applied in the Bayesian analysis was GTR+I+G, lset nst = 6, rates = invgamma and prset statefreqpr = dirichlet (1, 1, 1, 1). Bayesian analysis resulted in a nearly congruent topology with an average standard deviation of split frequencies = 0.007133 to ML analysis and, thus, only the ML tree is provided (Fig. 1).

The phylogeny (Fig. 1) included 28 different genera in *Perenniporia* s.l., of which have eight uncertain species in regard to the generic status without typical characteristics, viz. *P.eugeissonae* P. Du & Chao G. Wang, *P.luteola* B.K. Cui & C.L. Zhao, *P.nonggangensis* F.C. Huang & Bin Liu, *P.pseudotephropora* Chao G. Wang & F. Wu, *P.rosicola*, *P.straminea* (Bres.) Ryvarden, *P.subtephropora* B.K. Cui & C.L. Zhao and *P.tephropora*. Thus, they were adopted in *Perenniporia* temporarily and distinguished from *Perenniporia* s.s.

*Perenniporiaprunicola* nested in the *Perenniporis* s.s. clade and formed an independent lineage in the phylogeny (Fig. 1). In addition, it is related to *P.medulla-panis*, *P.substraminea* and *P.hainaniana*, these four species being addressed into the *Perenniporia* s.s. clade. Though *Perenniporiarosicola* grouped with four species of *Perenniporia* s.s. in a joint subclade, but without support. The sequences of *Crassisporusminutus* and *C.mollissimus* were obtained from holotypes and they nested in the genus *Crassisporus*.

ITS sequences produced significant alignments in NCBI (https://www.ncbi.nlm.nih.gov/) about *Perenniporiaprunicola*, the top ten of which represent *P.medulla-panis* and the similarities of them were less than 95%. The same goes for *P.rosicola*, the similarities of the top ten ITS sequences in NCBI were less than 90% excepting one sequence tagged *P.dendrohyphidia* (Zhou 273). They are consistent with our phylogeny.

### ﻿Taxonomy

#### ﻿ Perenniporia prunicola

Taxon classificationFungiPolyporalesPolyporaceae

Y.C. Dai, Yuan Yuan & Chao G. Wang sp. nov.

CBE4481A-A3F6-501A-A47C-443C8131CEF0

MycoBank No: 851532

Figs 2 , 3

##### Holotype.

China. Yunnan Province, Zhaotong, Yiliang County, Xiaocaoba Town, on living tree of *Prunus*, 2.IV.2023, Dai 24751 (BJFC040388).

**Figure 2.**
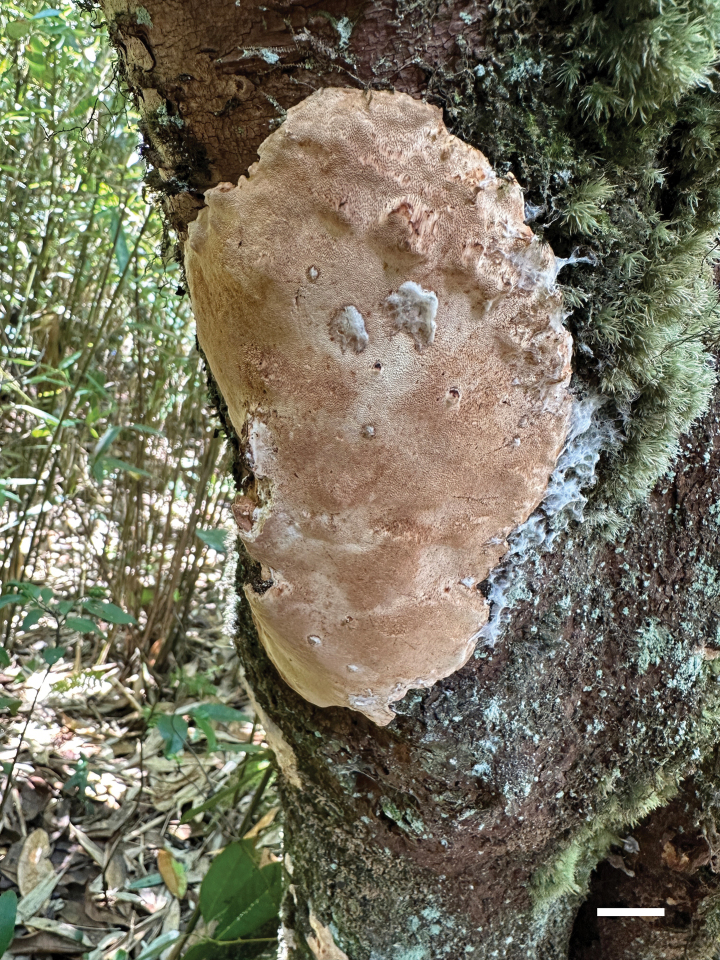
Basidiomata of *Perenniporiaprunicola* (Holotype, Y.C. Dai 24751). Scale bar: 1 cm.

##### Etymology.

*Prunicola* (Lat.): refers to the species growing on *Prunus*.

**Figure 3.**
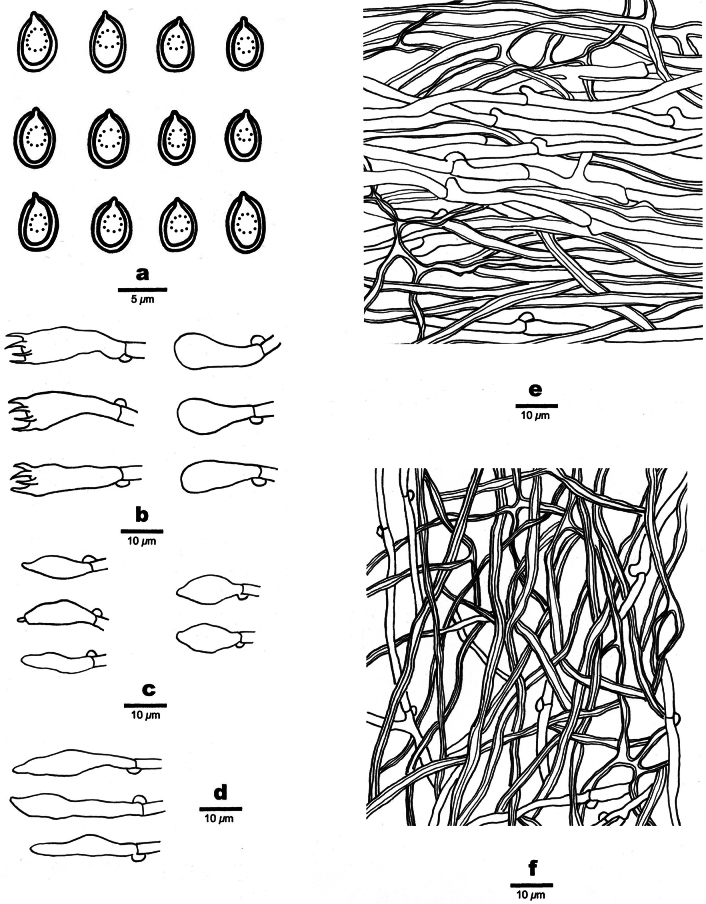
Microscopic structures of *Perenniporiaprunicola* (Holotype, Y.C. Dai 24751) **a** basidiospores **b** basidia and basidioles **c** cystidioles **d** hymenial cystidia **e** hyphae from subiculum **f** hyphae from trama.

##### Description.

***Basidiomata.*** Perennial, resupinate, corky, without odour or taste when fresh, becoming hard corky upon drying, up to 15 cm long, 5 cm wide and 16 mm thick at centre. Pore surface clay pink when fresh, becoming cream, buff yellow to fawn upon drying; sterile margin very narrow to almost absent; pores round to slightly elongated, 4–6 per mm; dissepiments slightly thick, entire. Subiculum thin, cream, corky, up to 1 mm thick. Tubes pinkish-buff to clay buff when dry, distinctly stratified, hard corky, up to 15 mm long.

***Hyphal structure.*** Hyphal system trimitic; generative hyphae bearing clamp connections; skeletal and binding hyphae IKI−, weakly CB+; tissues becoming orange brown in KOH.

***Subiculum.*** Generative hyphae frequent, hyaline, thin-walled, occasionally branched, more or less flexuous, 2–4 μm in diam.; skeletal hyphae dominant, hyaline, thick-walled with a wide lumen, occasionally branched, more or less flexuous, 2.5–3 μm in diam.; binding hyphae hyaline, thick-walled with a wide lumen, frequently arboriform branched, flexuous, interwoven, 1.5–2 μm in diam.

***Tubes.*** Generative hyphae infrequent, hyaline, thin-walled, occasionally branched, straight, 2–3 μm in diam.; skeletal hyphae dominant, hyaline, thick-walled with a medium lumen, occasionally branched, slightly flexuous, interwoven, 2–2.5 μm in diam.; binding hyphae hyaline, thick-walled with a medium lumen, frequently arboriform branched, flexuous, interwoven, 1.2–1.5 μm in diam. Hymenial cystidia present, clavate to fusiform, thin-walled, smooth, 25–31 × 5–5.5 µm; cystidioles present, ventricose to fusiform, hyaline, thin-walled, 16–20 × 4.5–5 μm. Basidia clavate, with four sterigmata and a basal clamp connection, 15–22 × 7–8 μm; basidioles more or less pyriform, but smaller. Irregular crystals present among the hymenium.

***Spores.*** Basidiospores ellipsoid to broadly ellipsoid, hyaline, thick-walled, smooth, usually with a medium guttule, dextrinoid, weakly CB+, (4.5–)4.8–6.2(–6.5) × (3.5–)3.6–4.5(–4.9) µm, L = 5.39 μm, W = 4.07 μm, Q = 1.29–1.37 (n = 90/3).

***Type of rot.*** White rot.

##### Additional specimens examined.

China. Guizhou Province, Zunyi, Suiyang County, Kuankuoshui Nature Reserve, on fallen trunk of *Prunus*, 7.VII.2022, Y.C. Dai 24280 (BJFC039522); Yunnan Province, Zhaotong, Yiliang County, Xiaocaoba, on dead tree of *Prunus*, 2.IV.2023, Y.C. Dai 24752 (BJFC040389).

##### Notes.

*Perenniporiaprunicola* is characterised by perennial and resupinate basidiomata with a clay pink pore surface when fresh, round to slightly elongated pores of 4–6 per mm, a trimitic hyphal system, the presence of clavate to fusiform hymenial cystidia, ellipsoid to broadly ellipsoid and thick-walled basidiospores measuring 4.8–6.2 × 3.6–4.5 µm and growth on *Prunus* in southwest China.

#### ﻿ Perenniporia rosicola

Taxon classificationFungiPolyporalesPolyporaceae

Y.C. Dai, Yuan Yuan & Chao G. Wang sp. nov.

1F1D717F-01AC-587D-9899-AB3F819BF8CB

MycoBank No: 851529

Figs 4 , 5

##### Holotype.

China. Yunnan Province, Mengla County, Xishuangbanna Rainforest Valley, on branch of Rosaceae, 4.VII.2021, Y.C. Dai 22563 (BJFC037137).

**Figure 4.**
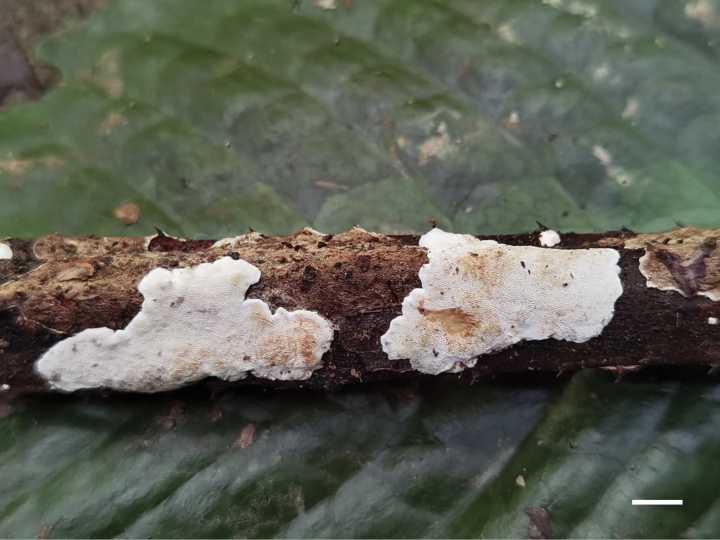
Basidiomata of *Perenniporiarosicola* (Holotype, Y.C. Dai 22563). Scale bar: 1 cm.

##### Etymology.

*Rosicola* (Lat.): refers to the species growing on Rosaceae.

**Figure 5.**
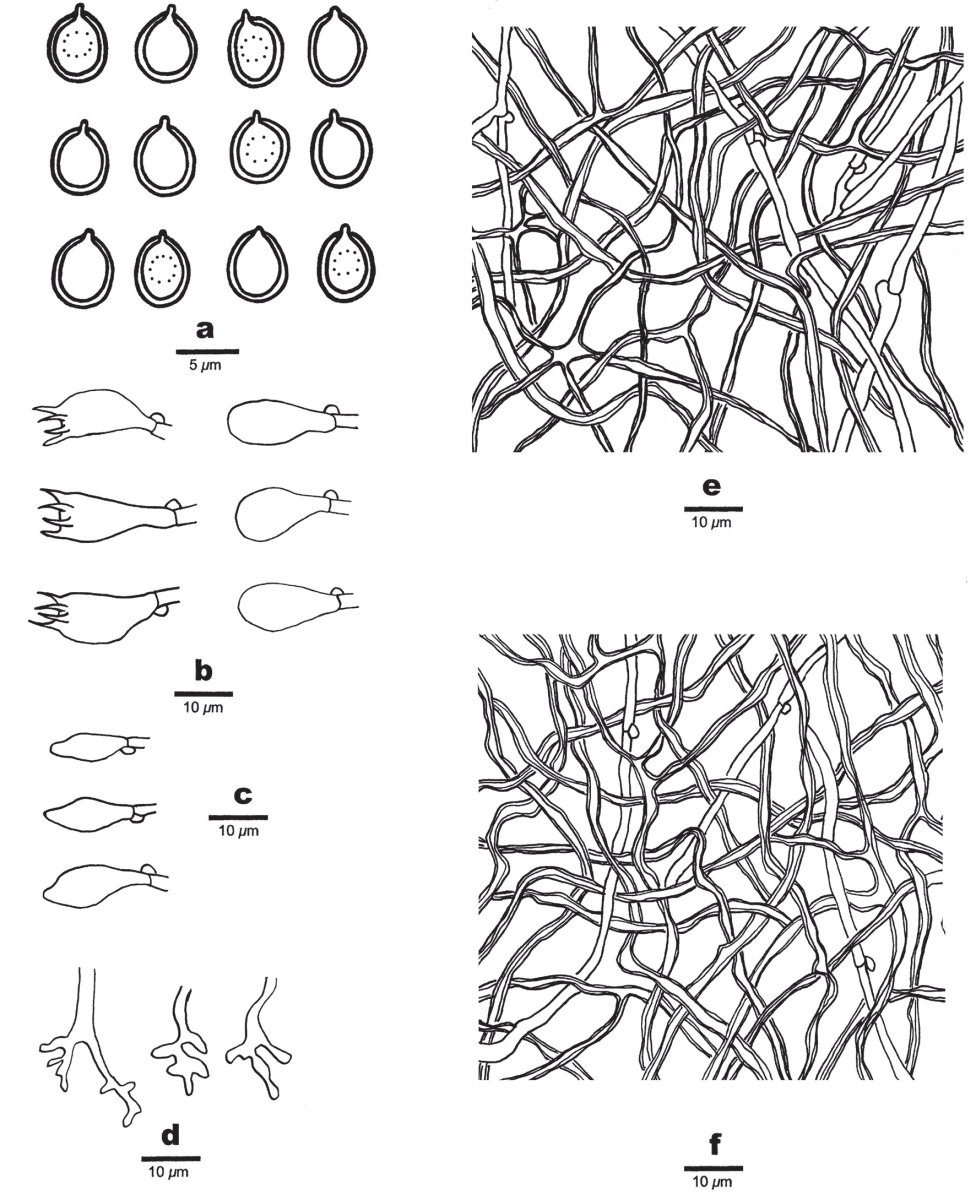
Microscopic structures of *Perenniporiarosicola* (Holotype, Y.C. Dai 22563) **a** basidiospores **b** basidia and basidioles **c** cystidioles **d** dendrohyphidia **e** hyphae from subiculum **f** hyphae from trama.

##### Description.

***Basidiomata.*** Annual, resupinate, soft corky, without odour or taste when fresh, becoming corky when dry, up to 2 cm long, 1.5 cm wide and 1.2 mm thick at centre. Pore surface white when fresh, becoming pale orange brown upon bruising, eventually honey yellow to clay buff upon drying; sterile margin white when fresh, becoming cream upon drying, up to 0.5 mm wide; pores round, sometimes elongated, 5–7 per mm; dissepiments thin, entire to slightly lacerate. Subiculum very thin, cream, corky, up to 0.2 mm thick. Tubes concolorous with pore surface, corky, up to 1 mm long.

***Hyphal structure.*** Hyphal system dimitic; generative hyphae bearing clamp connections; skeletal hyphae dextrinoid, weakly CB+; tissues becoming pale olivaceous in KOH.

***Subiculum.*** Generative hyphae infrequent, hyaline, thin-walled, occasionally branched, straight, 2–2.5 μm in diam.; skeletal hyphae dominant, hyaline, thick-walled with a medium to narrow lumen, frequently arboriform branched, flexuous, interwoven, 1.5–2.5 μm in diam.

***Tubes.*** Generative hyphae infrequent, hyaline, thin-walled, more or less flexuous, 2–2.5 μm in diam.; skeletal hyphae dominant, hyaline, thick-walled with a medium lumen, frequently arboriform branched, flexuous, interwoven, 1.5–2.5 μm in diam. Hymenial cystidia absent; cystidioles present, ventricose to fusiform, hyaline, thin-walled, 14–16 × 5–5.5 μm. Basidia barrel-shaped, with four sterigmata and a basal clamp connection, 16–20 × 7–8 μm; basidioles in shape similar to basidia, but smaller. Irregular crystals present amongst hymenia. Dendrohyphidia present.

***Spores.*** Basidiospores broadly ellipsoid to subglobose, hyaline, thick-walled, smooth, sometimes with a medium guttule, dextrinoid, weakly CB+, 5–5.8(–6) × 4–5.2(–5.3) µm, L = 5.39 μm, W = 4.74 μm, Q = 1.14 (n = 30/1).

***Type of rot.*** White rot.

##### Notes.

*Perenniporiarosicola* is characterised by annual and resupinate basidiomata with a white pore surface when fresh, round to sometimes elongated pores of 5–7 per mm, frequently arboriform branched and narrow skeletal hyphae, the presence of dendrohyphidia, broadly ellipsoid to subglobose, thick-walled basidiospores measuring 5–5.8 × 4–5.2 μm and growth on Rosaceae in southwest China.

### ﻿Combinations

In our phylogenetic analyses, *Crassisporusminutus* and *C.mollissimus* form two independent lineages nested in *Crassisporus* (Fig. 1) and their characteristics fit the definition of *Crassisporus*. So, we propose the following combinations:

#### ﻿ Crassisporus minutus

Taxon classificationFungiPolyporalesPolyporaceae

(Y.C. Dai & X.S. Zhou) Y.C. Dai, Yuan Yuan & Chao G. Wang comb. nov.

203C7214-61D2-5D42-BD86-22C820C37F19

MycoBank No: 851530

##### Basionym.

*Megasporoporiaminuta* Y.C. Dai & X.S. Zhou, in Zhou & Dai, Mycological Progress 7(4): 254 (2008).

#### ﻿ Crassisporus mollissimus

Taxon classificationFungiPolyporalesPolyporaceae

(B.K. Cui & C.L. Zhao) Y.C. Dai, Yuan Yuan & Chao G. Wang comb. nov.

E5518059-F446-52A7-988B-39B257D026B7

MycoBank No: 851531

##### Basionym.

*Abundisporusmollissimus* B.K. Cui & C.L. Zhao, in Zhao, Chen, Song & Cui, Mycological Progress 14(38): 5 (2015).

## ﻿Discussion

The genus *Perenniporia* s.s. clade includes four species, viz. *P.hainaniana*, *P.medulla-panis*, *P.prunicola* and *P.substraminea* and these species have the perennial and resupinate basidiomata with a cream, clay pink, buff yellow, pinkish-buff to fawn pore surface, a dimitic to trimitic hyphal system with amyloid or dextrinoid skeletal hyphae, ellipsoid, broadly ellipsoid to subglobose and thick-walled basidiospores (Table 2).

**Table 2.** The list of accepted species related to new taxa in this study.

Species	Type locality	Basidiomata	Upper surface	Colour of poroid surface	Dendrohyphidia	Basidiospores shape	Basidiospores size (μm)	References
*Crassisporusimbricatus*	China: Hainan	Annual, effused-reflexed to pileate	Yellowish-brown	Buff when fresh, pale greyish-brown when dry	–	Oblong ellipsoid	10–14 × 4.5–6.2	Ji et al. (2019)
*C.leucoporus*	Australia: Queensland	Annual, effused-reflexed to pileate	Yellowish-brown to umber-brown	White when fresh; cream, clay buff to pale yellowish-brown when dry	–	Oblong ellipsoid	8.4–11.2 × 4.2–5.4	Ji et al. (2019)
*C.macroporus*	China: Guangxi	Annual, effused-reflexed to pileate	Buff to yellowish-brown when fresh, yellowish brown when dry	Cream, buff to cinnamon buff when fresh; buff, pale yellowish-brown to yellowish-brown when dry	–	Oblong ellipsoid	9.5–13.2 × 4–6.2	Ji et al. (2019)
*C.microsporus*	China: Yunnan	Annual, pileate	Pale yellowish-brown to yellowish-brown	Cream, buff to cinnamon buff when fresh; buff, pale yellowish-brown to yellowish-brown when dry	–	Broadly ellipsoid	4–5 × 3–3.7	Ji et al. (2019)
***C.minutus***	China: Guangxi	Annual to biennial, resupinate	–	Cream to pale buff when fresh; pale greyish when dry	–	Cylindrical to oblong ellipsoid	7.7–9.7 × 3.6–4.9	Zhou and Dai (2008)
***C.mollissimus***	China: Hainan	Perennial, effused-reflexed to pileate	Yellow brown to umber-brown	Buff to buff-yellow when fresh, buff-yellow when dry	–	Ellipsoid	4–4.5 × 3–3.5	Zhao et al. (2015)
*Perenniporiaadnata*	Singapore	Perennial, resupinate	–	Ochraceous buff to pinkish ochraceous	–	Broadly ellipsoid to subglobose	4–4.5 × 3.5	Corner (1989)
*P.albocinnamomea*	Malaysia	Annual, effused-reflexed	Pallid buff to brownish	Light cinnamon buff	–	Ellipsoid	3.7–4.7 × 2.5–3	Corner (1989)
*P.dendrohyphidia*	Burundi	Annual, resupinate	–	Wood-coloured to pale isabelline	＋	Broadly ellipsoid to subglobose, sometimes truncate	5.5–7 × 4.5–6	Ryvarden (1988a), this study
*P.eugeissonae*	Malaysia	Annual, resupinate	–	White when fresh, cream to pale straw-coloured when dry	＋	Ellipsoid	5–6 × 4–5	Du et al. (2020)
*P.ferruginea*	Brunei	Perennial, effused-reflexed	Ferruginous brown to fuscous blackish	Pallid wood white to pale brown	–	Ellipsoid, subtriangular to subglobose	3.5–4.5 × 3–3.5	Corner (1989)
*P.hainaniana*	China: Hainan	Perennial, resupinate	–	Cream when fresh, cream-buff when dry	＋	Broadly ellipsoid, truncate	4–4.5 × 3–4	Zhao and Cui (2013a)
*P.luteola*	China: Henan	Annual, resupinate	–	Cream to buff when fresh, buff to yellowish-buff when dry	–	Ellipsoid, truncate	6.1–7 × 5–5.7	Zhao and Cui (2013b)
*P.medulla-panis*	Australia	Annual to perennial, resupinate	–	White when fresh; white, cream, pale corky when dry; greyish-orange when bruised	–	Ellipsoid, broadly ovoid to subglobose, truncate	4.7–5.8 × 3.5–4.5	Decock and Stalpers (2006)
*P.nonggangensis*	China: Guangxi	Annual, resupinate to effused-reflexed	–	Cream to greyish-cream when fresh; pale yellow-orange, capucine buff to sudan brown when dry	–	Broadly ellipsoid to subglobose	3.1–4.4 × 2.7–3.6	Huang et al. (2017)
*P.puerensis*	China: Yunnan	Annual, resupinate	–	Cream to buff when fresh, yellow to ochraceous when dry	–	Ovoid to subglobose	4.3–5.5 × 3.7–4.7	Liu et al. (2017)
*P.penangiana*	Malaysia	Annual, pileate with a stipe	Pale ochraceous to brownish	Pale tan ochraceous	–	Broadly ellipsoid	5–6.5 × 4–5	Corner (1989)
***P.prunicola***	China: Yunnan	Perennial, resupinate	–	Clay pink when fresh; cream, buff yellow to fawn when dry	–	Ellipsoid to broadly ellipsoid	4.8–6.2 × 3.6–4.5	This study
*P.pseudotephropora*	Brazil	Perennial, effused-reflexed to pileate	Pinkish buff, grey to greyish -brown	Greyish to pale brown	＋	Broadly ellipsoid to subglobose, truncate	4.9–5.2 × 4–4.8	Wang et al. (2020)
***P.rosicola***	China: Yunnan	Annual, resupinate	–	White when fresh; pale orange brown when bruised, eventually honey yellow to clay buff when dry	＋	Broadly ellipsoid to subglobose	5–5.8 × 4–5.2	This study
*P.sinuosa*	Brazil	Annual, resupinate	–	Cream to ochraceous	–	Subglobose, truncate	4–5 × 3–4	Ryvarden (1987)
*P.straminea*	Philippines	Annual, resupinate	–	Straw-coloured when fresh; pale yellow brown with orange tints when dry	–	Ellipsoid	2.5–3 × 2	Ryvarden (1988b)
*P.subdendrohyphidia*	Cameroon	Annual to biennial, resupinate	–	White, yellowish to pale pinkish cork-coloured when bruised	＋	Oblong, Oblong ellipsoid to ellipsoid, truncate	4–4.8 × 2.8–3.3	Decock (2001)
*P.substraminea*	China: Zhejiang	Perennial, resupinate	–	White to cream when fresh, cream to pinkish-buff when dry	＋	Ellipsoid, truncate	3.1–3.8 × 2.4–3	Zhao et al. (2013a)
*P.subtephropora*	China: Guangdong	Perennial, resupinate	–	Cream when fresh; cream buff to greyish-buff when dry	–	Ellipsoid to broadly ellipsoid, truncate	4–5 × 3.5–4.5	Zhao and Cui (2013a)
*P.tephropora*	Suriname	Perennial, resupinate to rarely effused-reflexed	Dirty greyish to black	Clay buff, grey to milky coffee or pale umber	–	Broadly ellipsoid, truncate	4.5–6 × 3.5–4.5	Ryvarden (1972b)

**Bold** = new taxa. Abbreviations used: + = Present, – = Absent.

*Perenniporiaprunicola* is similar to *P.medulla-panis* by perennial and resupinate basidiomata with a clay pink to buff yellow pore surface, round to slightly elongated pores of 4–6 per mm, a trimitic hyphal system and ovoid to broadly ellipsoid basidiospores. In addition, both species are phylogenetically related, but the latter lacks cystidia and usually has truncate basidiospores (Ryvarden and Gilbertson 1994). *Perenniporiapuerensis* C.L. Zhao has annual and thin basidiomata, thin dissepiments, thick-walled skeletal hyphal encrusted with pale yellow crystals, the absence of hymenial cystidia and relatively smaller basidiospores (4.3–5.5 × 3.7–4.7 µm vs. 4.8–6.2 × 3.6–4.5 µm; Q = 1.14–1.21 (n = 120/4) vs. Q = 1.29–1.37 (n = 90/3), Liu et al. (2017)), which differ from *P.prunicola*.

*Perenniporiarosicola* is morphologically similar and phylogenetically related to Perenniporiacf.dendrohyphidia (Fig. 1). We studied the type of *P.dendrohyphidia* (Rammeloo 6286) and they all have annual and resupinate basidiomata, the presence of dendrohyphidia and broadly ellipsoid to subglobose and thick-walled basidiospores. However, *P.dendrohyphidia* has thick and entire dissepiments, round pores of 4–6 per mm, sometimes apically truncate and relatively larger basidiospores (5.5–7 × 4.5–6 µm vs. 5–5.8 × 4–5.2 μm) and it occurs in Burundi, central Africa. Unfortunately, we did not obtain sequences from the type specimen of *P.dendrohyphidia*. We also studied the specimen of labelled Zhou 273 collected in China and it has thin and entire dissepiments, round to slightly elongated pores of 6–8 per mm, branched skeletal hyphae measuring 1.5–3.2 µm in diam., broadly ellipsoid to subglobose basidiospores measuring 5–6 × 4–5 µm. These characteristics are somewhat similar to *P.dendrohyphidia*. Thus, for the time being, we treat the specimen Zhou 273 as Perenniporiacf.dendrohyphidia. In addition, there are 20 base pairs differences between Perenniporiacf.dendrohyphidia and *P.rosicola*, which amounts to > 3% nucleotide differences in the ITS regions. *Perenniporiasubdendrohyphidia* Decock was originally described by Decock from Cameroon, central Africa. However, it has smaller, oblong to oblong-ellipsoid and non-dextrinoid basidiospores (4–4.8 × 2.2–3.3 µm vs. 5–5.8 × 4–5.2, Decock (2001)). *Perenniporiasinuosa* Ryvarden was originally described from Amazonas, Brazil (Ryvarden 1987) and it differs from *P.rosicola* by larger pores (2–3 per mm vs. 5–7 per mm) and smaller truncate basidiospores (4–5 × 3–4 µm vs. 5–5.8 × 4–5.2 µm, Ryvarden (1987)). *Perenniporiaadnata* Corner, *P.albocinnamomea* Corner, *P.ferruginea* Corner and *P.penangiana* Corner were all originally described from Southeast Asia and lack dendrohyphidia. In addition, the former three species above differ from *P.rosicola* by smaller basidiospores (4–4.5 × 3.5 µm in *P.adnate*; 3.7–4.7 × 2.5–3 µm in *P.albocinnamomea*; 3.5–4.5 × 3–3.5 µm in *P.ferruginea* vs. 5–5.8 × 4–5.2 µm, Corner (1989)). *Perenniporiapenangiana* has pileate basidiomata with a stipe, which is different from *P.rosicola* (Corner 1989).

All species in the *Perenniporia* s.s. clade have perennial basidiomata with a cream, clay pink, buff yellow, pinkish-buff to fawn pore surface, a dimitic to trimitic hyphal system, sometimes the presence of dendrohyphidia and truncate basidiospores. Perenniporiacf.dendrohyphidia and *P.rosicola* both have annual basidiomata with a white to cream pore surface, a dimitic hyphal system, the presence of dendrohyphidia and broadly ellipsoid to globose basidiospores without truncation. All in all, some morphological characteristics of above taxa are overlapping, but the *Perenniporia* s.s. clade is unrelated to the *Perenniporiarosicola* clade in our phylogeny (Fig. 1).

*Crassisporusminutus* was originally described in *Megasporoporia* by Dai and Zhou from China and it is characterised by resupinate basidiomata with a cream to pale buff pore surface when fresh, distinct sterile margin, round pores of 4–6 per mm, a dimitic hyphal system; thick-walled to subsolid skeletal hyphae, cylindrical to oblong-ellipsoid basidiospores measuring 7.7–9.7 × 3.6–4.9 µm (Zhou and Dai 2008). The type specimen of *M.minutus* Zhou 120 grouped with other samples Dai 22571 and Cui 6595 nested in *Crassisporus* in our phylogenetic analysis (Fig. 1). However, we studied the sample Dai 22571 and it has slightly thick-walled basidiospores. Thus, the new combination *Crassisporusminutus* is proposed.

*Crassisporusmollissimus* was originally described in *Abundisporus* by Cui and Zhao from China and it is characterised by perennial, effused-reflexed to pileate basidiomata with a concentrically zonate pileal surface, a buff to buff yellow pore surface when fresh, round pores of 7–8 per mm, ellipsoid and slightly thick-walled basidiospores measuring 4–4.5 × 3–3.5 µm (Zhao et al. 2015). In addition, *Crassisporus* and *Abundisporus* are phylogenetically unrelated (Fig. 1).

## Supplementary Material

XML Treatment for Perenniporia prunicola

XML Treatment for Perenniporia rosicola

XML Treatment for Crassisporus minutus

XML Treatment for Crassisporus mollissimus

## ﻿Additional information

### Author ORCIDs

Chao-Ge Wang https://orcid.org/0000-0003-4381-5720

Yuan Yuan https://orcid.org/0000-0001-6674-9848

### Data availability

All of the data that support the findings of this study are available in the main text.
